# Surface Modification of CaCO_3_ by Ultrasound-Assisted Titanate and Silane Coupling Agents

**DOI:** 10.3390/ma16103788

**Published:** 2023-05-17

**Authors:** Peng Cheng, Lei Yang, Yuxiong Xie, Yu Liu

**Affiliations:** 1 School of Civil Engineering, Nanyang Institute of Technology, Nanyang 473004, China; 2 Shaanxi Key Laboratory of Environmental Engineering, Xi’an University of Architecture and Technology, Xi’an 710055, China

**Keywords:** CaCO_3_, surface modification, silane coupling agent, titanate, ultrasonication

## Abstract

Calcium carbonate (CaCO_3_) is a widely used inorganic powder, but its industrial applications are limited by its hydrophilicity and oleophobicity. Surface modification of CaCO_3_ can improve its dispersion and stability in organic materials and further improve its potential value. In this study, CaCO_3_ particles were modified with silane coupling agent (KH550) and titanate coupling agent (HY311) combined with ultrasonication. The oil absorption value (OAV), activation degree (AG), and sedimentation volume (SV) were employed to evaluate the modification performance. The results showed that the modification effect of HY311 on CaCO_3_ was better than that of KH550, and ultrasonic treatment played an auxiliary role. Based on response surface analysis, the optimal modification conditions were determined as follows: the HY311 dosage was 0.7%, the KH550 dosage was 0.7%, and ultrasonic time was 10 min. The OAV, AG, and SV of modified CaCO_3_ under these conditions were 16.65 g DOP/100 g, 99.27%, and 0.65 mL/g, respectively. The SEM, FTIR, XRD and thermal gravimetric analyses indicated successful coating of HY311 and KH550 coupling agents on the surface of CaCO_3_. The optimization of the dosages of two coupling agents and ultrasonic time improved the modification performance significantly.

## 1. Introduction

As an inorganic raw material, calcium carbonate (CaCO_3_) is one of the cheapest and most abundant minerals found on Earth [1,2]. It is widely used as filler material in the field of rubber, plastics, papermaking, coatings [3,4], etc. Small CaCO_3_ particles with hydrophilic and oleophobic properties are generally prone to agglomerate, leading to poor dispersion in polymer matrix [5,6]. Therefore, to improve the application potential of CaCO_3_ in industries, it is necessary to modify the surface of CaCO_3_ to improve its affinity and dispersion stability in hydrophobic polymers. The surface of CaCO_3_ is commonly modified with hydrophobic surfactants [7], such as silane coupling agents [8,9], titanate coupling agents [10], aluminate coupling agents [11], fatty acids [12], etc. However, the modification performance is still expected to be improved to achieve better dispersion and compatibility in the polymer matrix and widen the potential functionalization of CaCO_3_ [13,14].

Silane coupling agents are commonly used for the surface modification of many inorganic powders, such as talcum powder, aluminum hydroxide, and magnesium hydroxide [15,16]. The surface of copper (Cu) powder was modified with 3-aminopropyltriethoxysilane (KH550) to improve its corrosion-resistant property [17]. Robaidi et al. [18] modified the CaCO_3_ surface with a silane coupling agent, which improved the dispersion of CaCO_3_ in a polyvinyl chloride (PVC) matrix and also provided good interfacial adhesion, along with high tensile and impact strengths to the calcium carbonate/PVC composites. Yang et al. [8] used a silane coupling agent to modify the surface of CaCO_3_ nanoparticles, by which the interfacial compatibility between CaCO_3_ nanoparticles and styrene-butadiene rubber (SBR) latex was significantly improved.

It was reported that titanate coupling agents also have an obvious improvement on both inorganic filler dispersion and inorganic–organic interface conditions [19]. Latinwo et al. [20] modified calcium carbonate with a titanate coupling agent in isopropanol solvent, which enhanced its dispersion and interfacial bonding in polyurethane foam and also increased the tensile strength of polyurethane foam. Cheng et al. [21] modified the surface of pulverized coal with titanate and silane coupling agents by the ultrasonic wet method, and the modified pulverized coal could form a stable colloidal dispersion in anhydrous ethanol. Both silane and titanate coupling agents are amphoteric organic compounds with groups interacting with the surface of CaCO_3_ particles and organic polymer simultaneously [22]. One part of the group of molecules in the coupling agents can react with the –OH group on the surface of CaCO_3_ to form a strong chemical bond, and the other part of the group can establish covalent bonds with organic materials [1,13]. When the silane and titanate coupling agents dissolve and mix with the anhydrous ethanol, the organic moieties of the two coupling agents may interact with each other and improve the modification performance. Therefore, the combined application of silane and titanate coupling agents may achieve better modification performance on CaCO_3_.

In addition, ultrasonic treatment is also commonly employed in the modification process [23]. The ultrasonic (US) dispersion technique generates cavitation bubbles that locally produce high temperature and high pressure; the bubbles collapse or disappear in the liquid medium and produce a huge impact force and micro-jets [24]. The energy generated by ultrasonic cavitation improves the interactions between organic modifiers and inorganic particles. This facilitates the coating of the modifier on the surface of the inorganic powder. Ultrasonic treatment has achieved better dispersion of nano titanium carbide (TiC) powders aided by Tween 80 addition [25]. Hence, ultrasonic treatment can further improve the lipophilic and hydrophobic properties of CaCO_3_ in the modification process. However, to the best of our knowledge, there have been few reports on the modification of CaCO_3_ by ultrasound-assisted silane and titanate coupling agents.

Therefore, this study proposes and explores the effects of an ultrasound-assisted silane coupling agent (KH550) and a titanate coupling agent (HY311) on the modification of CaCO_3_. The main objectives of this study were: (1) to determine the effects of the KH550 and HY311 combined with or without ultrasonic treatment; (2) to optimize the treatment conditions of ultrasound-assisted KH550 and HY311 modification based on response surface methodology; and (3) to characterize the modified CaCO_3_ with scanning electron microscopy (SEM), Fourier transform infrared spectroscopy (FTIR), thermal gravimetric analysis (TGA), X-ray diffraction (XRD) and particle size analyzer.

## 2. Materials and Methods

### 2.1. Materials

CaCO_3,_ with an average particle size of 14.56 μm, was procured from Tianjin Deen Chemical Reagent Co., Ltd. (Tianjin, China). The titanate coupling agent (HY311, ethanediolato titanate) and silane coupling agent (KH550, 3-aminopropyltriethoxy silane) were sourced from Heyuan Chemical Co., Ltd. (Huaian, China) and Chuangshi Chemical Co., Ltd. (Nanjing, China), respectively.

### 2.2. Surface Modification of CaCO_3_

A certain amount of HY311 or KH550 was weighed and dissolved in anhydrous ethanol. Ethanol solution (15 mL) with different amounts of coupling agents (given in wt% based on CaCO_3_) were added into a beaker with 20 g of CaCO_3_. The mixtures were ultrasonicated at 90 W for 12 min at room temperature when the ultrasonic treatment was conducted. Then, the mixture was filtered and dried in an oven at 80 °C for 24 h. Thereafter, the modified CaCO_3_ product was obtained after grinding the residue.

### 2.3. Performance Test

#### 2.3.1. Oil Absorption Value

Oil absorption values (OAV) is the mass of dibutyl phthalate (DOP) adsorbed per 100 g CaCO_3_. OAV (g DOP/100 g) was tested according to the methods described by previous studies [26,27] and was calculated using Equation (1):

(1) $$\mathrm{OAV}=\frac{100\left(M_{3}-M_{2}\right)}{M_{1}}$$

where M_1_ (g) is the mass of modified CaCO_3_ powder, M_3_ (g) is the weight of the dropping bottle and DOP before DOP addition (g), and M_2_ (g) is the weight of the dropping bottle and DOP after DOP addition (g). DOP was added to the CaCO_3_ powder until all the particles clustered. All tests in this study were conducted in triplicate and the mean values were reported.

#### 2.3.2. Activation Degree 

Activation degree (AG) was determined according to the method described by Hu et al. [26] and Cao et al. [28]. AG (%) was obtained using Equation (2):

(2) $$\mathrm{AG}=\left[1-\frac{\left(M_{4}-M_{5}\right)}{M_{1}}\right]\;\times \;100\%$$

where M_1_ (g) is the mass of modified CaCO_3_ powder, M_5_ (g) is the mass of glass sand crucible, and M_4_ (g) is the mass of the uncoated CaCO_3_ and glass sand crucible after drying in the test.

#### 2.3.3. Sedimentation Volume

Sedimentation volume (SV, mL/g) was determined using Equation (3):

(3) $$\mathrm{SV}=\frac{V_{1}}{M_{1}}$$

where V_1_ (mL) is the volume of the sediment in the test. The modified CaCO_3_ particles (M_1_ g) were placed in a 50 mL measuring cylinder and liquid paraffin was added to the 50 mL mark. Then, the suspension mixture was fully stirred to distribute CaCO_3_ evenly in the liquid paraffin. After standing for 24 h, the volume of the sediment V_1_ (mL) was recorded.

### 2.4. Optimization of Modification Process

To enhance the modification performance and reduce the cost of chemicals, the combined modification of CaCO_3_ by ultrasound-assisted silane and titanate coupling agents was studied. Because both the amount of the coupling agents (HY311 and KH550) and the ultrasonication time (UST) may affect the modification performance of CaCO_3_, multivariate statistical models were used to understand the effect of one parameter and its role in finding the optimum point. Response surface analysis was employed to estimate the multivariate polynomial fitted with the independent variables using Design Expert software. The OAV, AG, and SV of the modified CaCO_3_ were tested and evaluated in a sequence of 17 experimental runs with appropriate combinations, including the dosage of HY311 (0.3–1.1%), the dosage of KH550 (0.3–1.1%) and ultrasonication time (8–12 min). Thereafter, experimental verification was conducted under optimum conditions.

### 2.5. Characterization

The particle sizes of CaCO_3_ before and after surface modification were measured using a laser particle size analyzer (LS230, Beckman Coulter, Brea, CA, USA). The functional groups of CaCO_3_ samples were characterized by Fourier transform infrared (FTIR, Nicolet iS50, Thermo Scientific, Waltham, MA, USA) in the range of 400–4000 cm^−1^ using the KBr disc method. The morphologies of the materials were obtained by scanning electron microscopy (SEM, Quanta FEG 250, FEI Corporation, Hilsboro, OR, USA). The thermal gravimetric analysis (TGA) was performed on a thermogravimetric analyzer (TGA55, TA Instrument, New Castle, DE, USA). The original and modified CaCO_3_ samples were heated from room temperature to 800 °C at a rate of 10 °C/min in a nitrogen atmosphere. The crystal structures of the CaCO_3_ before and after modification were examined by X-ray diffraction (XRD, Ultima IV, Rigaku Corporation, Tokyo, Japan) in the 2θ range of 5–90°.

## 3. Results and Discussion

### 3.1. Single-Factor Experiments

#### 3.1.1. Effect of KH550 Combined with Ultrasonic Treatment

**Figure 1:**
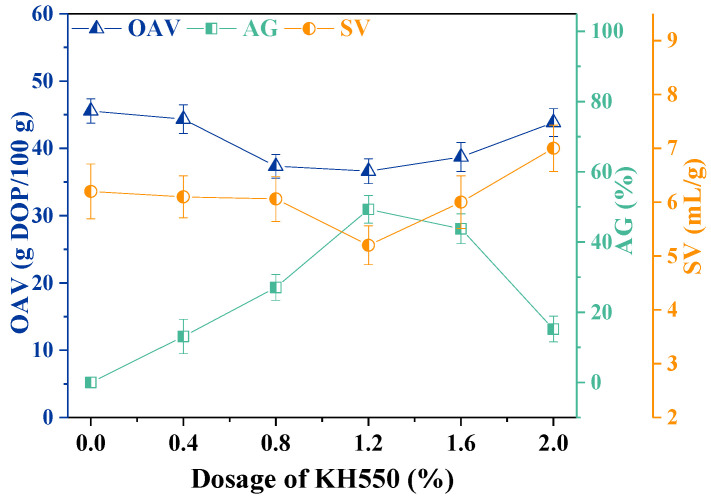
Effect of KH550 dosage combined with ultrasonic treatment.

The CaCO_3_ particles were first modified by the ultrasound-assisted (12 min) KH550 treatment. The OAV, AG, and SV values of the CaCO_3_ were used to evaluate the modification performance. Smaller OAV and higher AG indicate better CaCO_3_ nanoparticle processability and surface hydrophobicity [28,29]. The SV can also reflect the dispersion potential of particles in the liquid phase. As shown in Figure 1, the OAV, SV, and AG of unmodified CaCO_3_ were found to be 45.56 g DOP/100 g, 6.2 mL/g, and 0%, respectively. With increased KH550 dosage, OAV and SV decreased first and then increased, whereas AG exhibited an opposite trend.

When the KH550 dosage was 1.2% (given in wt% based on CaCO_3_), the OAV and SV of CaCO_3_ decreased to a minimum of 36.60 g DOP/100 g and 5.2 mL/g, respectively, whereas the AG reached a maximum of 49.29%. Hence, the hydrophobicity and dispersibility of the modified CaCO_3_ surface were the highest under these conditions. When the dosage of KH550 was under 1.2%, the surface of CaCO_3_ was not covered sufficiently [26]. Nevertheless, an excessive organic molecular chain with the KH550 dosage above 1.2% could increase the surface energy potential of modified CaCO_3_, strengthen its hydrophilicity, and increase the OAV value [22]. Meanwhile, the organic layer formed by the silane coupling agent improved the activation degree of CaCO_3_ significantly.

#### 3.1.2. Effect of HY311 Dosage without Ultrasonic Treatment

**Figure 2:**
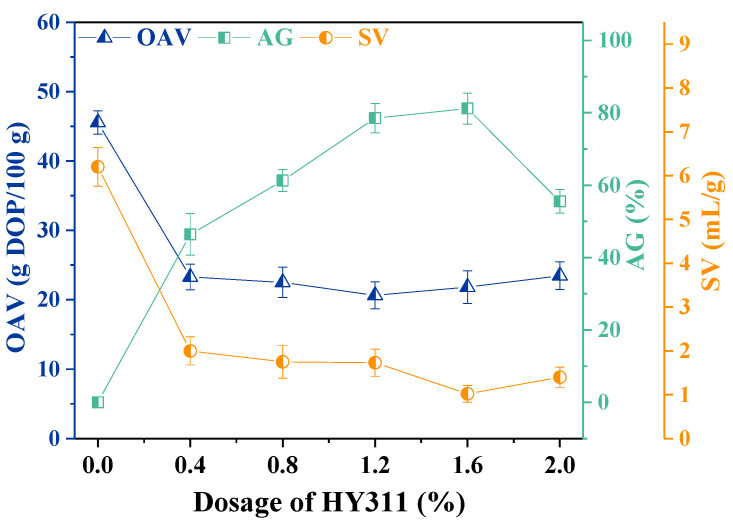
Effect of HY311 dosage on OAV, AG and SV of the modified CaCO_3_.

The effect of HY311 dosage on the OAV, AG, and SV of the modified CaCO_3_ are shown in Figure 2. With increased HY311 dosage, OAV and SV decreased significantly, whereas AG increased first and then decreased. When the amount of HY311 was 1.6%, AG reached a maximum of 81.17%, and SV decreased to the minimum value of 1.02 mL/g. Meanwhile, compared with the modification of CaCO_3_ with KH550, the modification performance of HY311 clearly improved.

When the HY311 dosage increased from 0.4% to 1.2%, the number of CaCO_3_ particles coated with HY311 gradually increased. This led to a slight decrease in OAV, which was consistent with the findings of Hu et al. [26]. Then, with further increase in the amount of HY311, the surface coating of CaCO_3_ particles reached its saturation, and thus, there was not much change in OAV.

When the HY311 dosage was increased to 1.6%, the lipophilic ends of the modifier molecules that were coated on the surface of CaCO_3_ particles extended outwards, rendering the particles insoluble in water [30], and the AG reached a maximum of 81.17%. The higher the degree of activation, the more hydrophobic the surface of CaCO_3_ particles [29]. This resulted in better dispersion of modified CaCO_3_ in liquid paraffin, and so the SV decreased to the minimum value of 1.02 mL/g.

With further increase in the addition of HY311, there was a slight change in SV and a significant decrease in AG. The reason for this could be that the amount of coupling agent added was too large; the surface of the CaCO_3_ showed a change from single-layer adsorption to double-layer or multi-layer adsorption. The hydrophilic ends of the excessive number of modifier molecules were directed outwards [26], which reduced the lipophilicity of the CaCO_3_ surface.

#### 3.1.3. Effect of HY311 Combined with Ultrasonic Treatment

**Figure 3:**
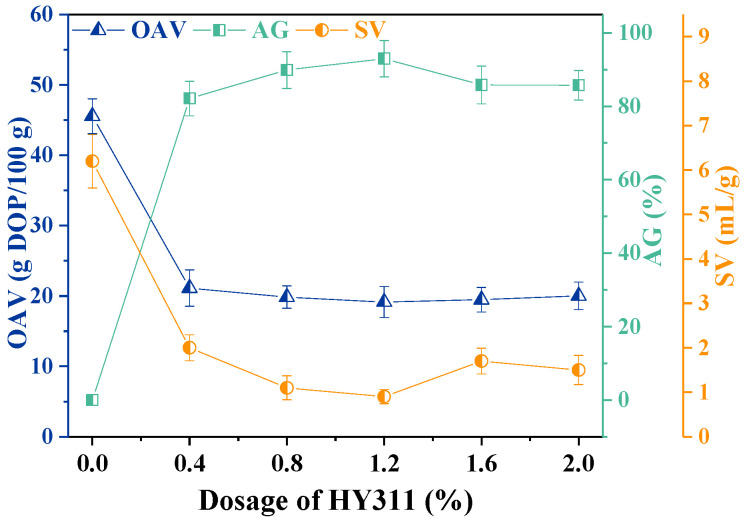
Effect of HY311 dosage combined with ultrasonic treatment.

The OAV, AG, and SV of CaCO_3_ modified by HY311 combined with ultrasonic treatment of 12 min are shown in Figure 3. With the increasing amount of HY311, the changes in OAV, AG, and SV showed similar trends compared to Figure 2. When the addition of HY311 was increased to 1.2%, OAV decreased from 45.56 g DOP/100 g to a minimum of 19.13 g DOP/100 g, AG increased from 0 to a maximum of 92.98%, and SV decreased from 6.2 mL/g to a minimum of 0.9 mL/g. The AG and SV indicated enhanced modification performance with ultrasonic treatment.

Figure 3 shows that 1.2% of HY311 had the best modification effect on CaCO_3_ when ultrasonicated for 12 min. When the amount of HY311 was more than 1.2%, OAV showed little change due to the single layer of modifier formed on the surface of CaCO_3_. However, excessive modifier molecules affected the dispersibility of CaCO_3_ particles in liquid paraffin and reduced AG, which could be explained by the decreased lipophilicity of the CaCO_3_ surface [13,22]. Compared with the modification of CaCO_3_ with HY311 individually, ultrasound-assisted dispersion could further increase the AG and reduce the OAV and SV of CaCO_3_, resulting in significantly better modification performance. Therefore, the treatment of CaCO_3_ by ultrasound-assisted silane and titanate coupling agents may further improve the modification performance.

### 3.2. Response Surface Analysis

**Figure 4:**
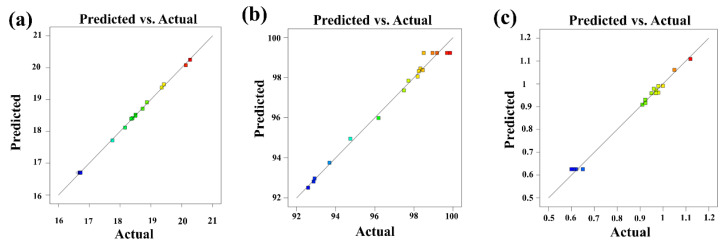
Correlations of model prediction on OAV (**a**), AG (**b**), and SV (**c**). The varying color of the squares from blue to red indicates the increase of the actual values.

In order to determine the optimal experimental conditions for the modification of CaCO_3_, a multi-factor experimental strategy was designed based on single-factor experiments. The experimental design and results with OAV, AG, and SV are shown in Table 1. The correlation between the experimentally determined real value and the predicted value of the model under the three-factor scheme is shown in Figure 4.

According to Figure 4a–c, the correlation coefficients (R^2^) between the predicted value and the real values of OAV, AG, and SV of modified CaCO_3_ were 0.9989, 0.9866, and 0.9926, respectively. The small differences between the predicted values and the real values indicated the good fit of the models. Taking the OAV, AG, and SV as the response values, the quadratic multinomial regression equations obtained by RSM tests are shown in the Supplementary Material as Equations (S1)–(S3). The analysis of variance of regression model equations are shown in Tables S1–S3. The *p* values of the models were all less than 0.001 (significant), indicating the high reliability of the models.

**Figure 5:**
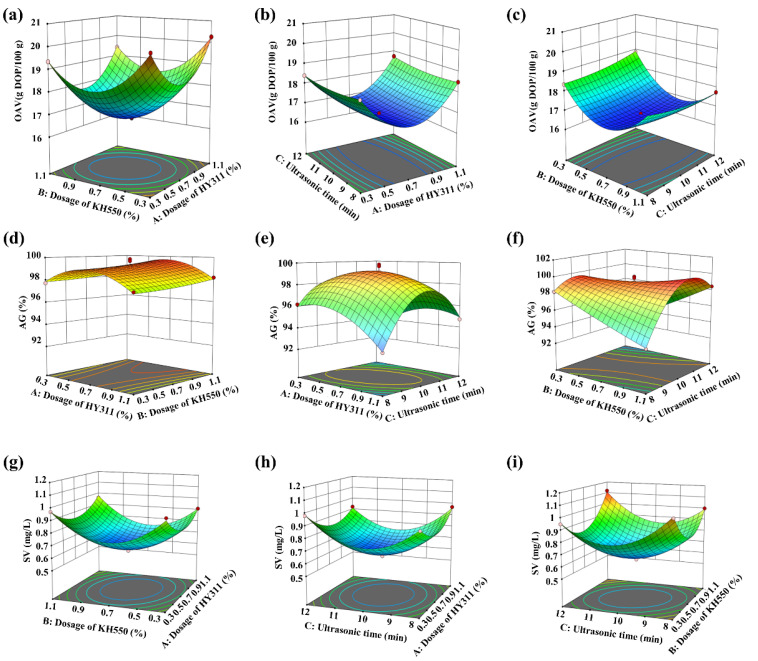
Response surface plots of OAV (**a**–**c**), AG (**d**–**f**), and SV (**g**–**i**) based on the effect of A (HY311 dosage), B (KH550 dosage) and C (ultrasonic time). The varying color of the curved surface from blue to red indicates the increase of the values. The circles represent the values obtained in the tests, which are red if they are above the predicted value and pink if they are below the predicted value.

As shown in Figure 5, the 3D-response surface clearly showed the changes in OAV, AG, and SV after the modification of CaCO_3_. In Figure 5a–c, the OAV of modified CaCO_3_ changed with the addition of HY311 and KH550 and the different UST. When the dosages of HY311 and KH550 were 0.7% with a UST of 10 min, the OAV of modified CaCO_3_ was minimum, and the effect of CaCO_3_ modification was the best. The order in which the three factors influenced the response value OAV was: HY311 > KH550 > UST. This was consistent with the results of single-factor tests, in which HY311 had the best modification effect on CaCO_3_ when ultrasonicated for 12 min.

It could be seen from Figure 5d–f and Figure 5g–i that when the UST was 10 min and the amount of HY311 and KH550 added were both 0.7%, the AG and SV of modified CaCO_3_ were the highest and lowest during the tests, respectively. Meanwhile, Figure 5a,d,g show that the combined use of two coupling agents dramatically improved the modification performance. Furthermore, the UST also had a significant effect on AG.

Based on the RSM results and the quadratic multinomial regression equations (Equation (S1)–(S3) in the Supplementary Material), the model achieved a minimum OAV of 16.73 g DOP/100 g. For this, the modification conditions were: UST of 9.45 min, 0.69% HY311, and 0.75% KH550. Similarly, when the model achieved the maximum value of AG (99.27%), UST was 9.87 min, HY311 dosage was 0.71%, and KH550 dosage was 0.67%. When the minimum value of SV reached 0.65 mL/g, the UST was 9.94 min, and HY311 and KH550 dosages were 0.71% and 0.68%, respectively.

Therefore, in this study, the best modification scheme of CaCO_3_ included a UST of 10 min and 0.7% dosages of HY311 and KH550. Under the optimal conditions for modification, experimental verification was carried out. The OAV of modified CaCO_3_ was 16.65 g DOP/100 g, AG was 99.27%, and SV was 0.65 mL/g. The error between experimental and predicted values was small, indicating that the results of response surface optimization were reliable.

### 3.3. Characterization of CaCO_3_ Particles

#### 3.3.1. Surface Morphology

**Figure 6:**
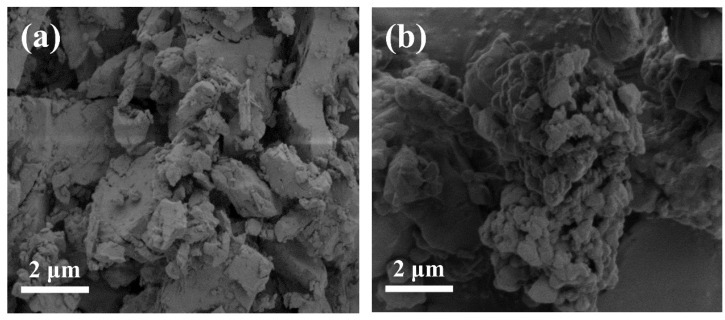
SEM images of (**a**) the original CaCO_3_ and (**b**) CaCO_3_ modified with KH550 and HY311 coupling agents combined with ultrasound treatment.

The morphology of CaCO_3_ is one of the properties for its industrial applications [31,32]. The SEM images of original CaCO_3_ and the CaCO_3_ modified using the optimal modification condition (the HY311 dosage was 0.7%, the KH550 dosage was 0.7%, and ultrasonic time was 10 min) are shown in Figure 6. As presented in Figure 6a, the surface of the unmodified CaCO_3_ particles were relatively flat and smooth, and most of them showed angular block structures. After modification, several irregular particles were adsorbed on the surface of the modified CaCO_3_. This could be due to the coating of CaCO_3_ surface with an organic layer during the modification process [28].

#### 3.3.2. Fourier Transform Infrared Spectra

**Figure 7:**
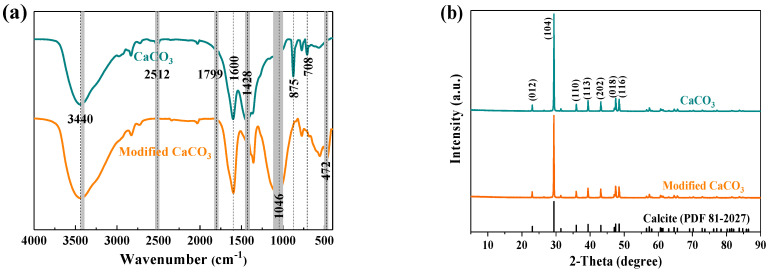
FTIR spectra (**a**) and XRD patterns (**b**) of CaCO_3_ before and after modification.

The FTIR spectra of CaCO_3_ before and after modification are shown in Figure 7a. The adsorption peaks at 3440 cm^−1^ and 1600 cm^−1^ could respectively be attributed to the stretching and bend vibrations of H–O–H, which might belong to the adsorbed water on the surface [33,34]. The absorption peaks of unmodified CaCO_3_ at 2512 and 1799 cm^−1^ could be attributed to the CO_3_^2-^ groups [2,14]. Peaks for antisymmetric stretching vibrations, out-of-plane bending vibrations, and in-plane bending vibrations of CO_3_^2-^ appeared also at 1428 [14,35], 875 [14,36], and 708 cm^−1^ [36,37], respectively.

After modification, the FTIR spectrum of CaCO_3_ showed a strong new absorption peak at 1046 cm^−1^, which was due to the antisymmetric stretching vibrations of the Si–O bond [38]. Meanwhile, the peak at 472 cm^−1^ could also be assigned to the stretching vibrations of the Si–O–Si bond [39]. This confirmed the successful coating of the silane coupling agent on the surface of CaCO_3_ particles. The peaks at 2512, 1799, 1428, 875, and 708 cm^−1^ in the spectrum of unmodified CaCO_3_ were dramatically changed in the spectrum of modified CaCO_3_, which either disappeared or decreased in intensity. These results indicated that the coupling agents were coated on the surface of the CaCO_3_.

#### 3.3.3. X-ray Diffraction Patterns and Thermogravimetric Analysis

The XRD pattern of original CaCO_3_ in Figure 7b is well-matched with the standard card profiles recorded on PDF 81-2027, which are the characteristic peaks of calcite. After modification with KH550 and HY311 coupling agents combined with ultrasound treatment, there were no obvious newly appeared peaks in the XRD patterns, indicating that the main crystal structure of CaCO_3_ was unchanged. Meanwhile, the intensity of the peaks was slightly weakened, which was due to the modifier molecules coated on the surface of the CaCO_3_ particles [14]. This result was also consistent with previous studies [2,27,29], which suggested the successful modification of CaCO_3_.

The TGA curves of the original and modified CaCO_3_ are presented in Figure S1 in the Supplementary Material. Before 550 °C, both the CaCO_3_ samples exhibited good thermal stability and almost no mass loss. At temperatures above 550 °C, mass losses of the original and modified CaCO_3_ particles were clearly observed, which might be mainly attributed to the thermal decomposition of CaCO_3_ to produce CaO and CO_2_ [14]. However, the decomposition temperature of modified CaCO_3_ was noticeably lower than that of the original CaCO_3_, which could be attributed to the thermal decomposition of the coupling agents. Similar results were also obtained in previous studies [2,8].

#### 3.3.4. Particle Size Analysis

**Figure 8:**
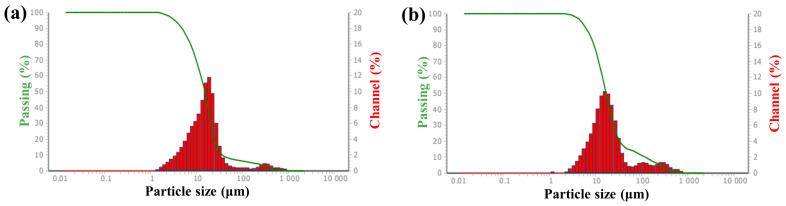
Particle size distribution of unmodified CaCO_3_ (**a**) and modified CaCO_3_ under the optimum modification conditions (**b**).

The particle size of CaCO_3_ affects both the filling effect and the cost in the application [13,40]. Particle size analysis was employed to determine the particle size distribution of CaCO_3_ particles and the effect of modification. The particle size distributions of unmodified CaCO_3_ and CaCO_3_ modified by the optimal conditions (UST: 10 min, HY311 dosages of 0.7% and KH550 dosages of 0.7%) are presented in Figure 8. The particle size distribution of unmodified CaCO_3_ was divided into two parts. The minimum and maximum particle sizes of CaCO_3_ were 1.16 μm and 704 μm, respectively. The particle sizes were mainly between 2–700 μm, with an average particle size of 14.56 μm. In Figure 8b, the minimum and maximum particle sizes of the modified CaCO_3_ increased to 1.95 μm and 837.2 μm, respectively. The particle sizes were mainly in the range of 3–800 μm with an average particle size of 89.15 μm.

Figure 8b showed that the particle size distribution of CaCO_3_ was only slightly affected by the modification. The particle diameters were mainly in the same range as the original CaCO_3_, despite the obviously improved OAV, AG, and SV of the modified CaCO_3_. Meanwhile, the average particle size increased from 14.56 to 89.15 μm, which could be due to the agglomeration of small particles and successful surface modification [41].

## 4. Conclusions

According to the OAV, AG, and SV of the modified CaCO_3_, the effect of HY311 modification on CaCO_3_ was better than that of KH550, and ultrasonic treatment played an auxiliary role. Based on response surface analysis, the optimal conditions for modification were found to be HY311 dosage of 0.7%, KH550 dosage of 0.7%, and UST of 10 min. In the tests under this condition, the actual OAV, AG, and SV of modified CaCO_3_ were 16.65 g DOP/100 g, 99.27%, and 0.65 mL/g, respectively. The optimization of the dosages of two coupling agents and UST improved the modification performance significantly. The SEM, FTIR, XRD and thermal gravimetric analyses indicated successful coating of HY311 and KH550 coupling agents on the surface of CaCO_3_.

## Figures and Tables

**Table 1:** Response surface design and results on the modification conditions.

Run No.	HY311 (%)	KH550 (%)	UST (min)	OAV (g DOP/100 g)		AG (%)		SV (mL/g)	
				Actual Value	Predicted Value	Actual Value	Predicted Value	Actual Value	Predicted Value
1	0.7	0.7	10	16.63	16.73	99.18	99.23	0.6100	0.6260
2	0.7	1.1	8	18.16	18.12	93.68	93.76	1	0.9911
3	0.7	0.3	8	18.36	18.39	98.25	98.32	1.0500	1.0600
4	0.3	0.7	8	18.50	18.52	96.19	95.99	0.9230	0.9305
5	1.1	0.7	12	18.73	18.71	94.75	94.95	0.9230	0.9155
6	1.1	0.3	10	20.27	20.25	97.49	97.36	0.9100	0.9086
7	0.7	1.1	12	17.75	17.72	98.45	98.38	1.1200	1.1100
8	1.1	1.1	10	19.42	19.47	98.19	98.06	0.9600	0.9786
9	0.3	0.3	10	20.13	20.08	97.72	97.85	0.9800	0.9614
10	0.7	0.7	10	16.68	16.73	98.50	99.23	0.6200	0.6260
11	0.7	0.7	10	16.62	16.72	99.85	99.23	0.6500	0.6260
12	0.7	0.7	10	16.71	16.73	99.69	99.23	0.6500	0.6260
13	0.3	1.1	10	19.35	19.37	98.33	98.45	0.9700	0.9714
14	0.7	0.3	12	18.87	18.91	92.59	92.51	0.9500	0.9589
15	0.7	0.7	10	16.72	16.73	98.95	99.23	0.6000	0.6260
16	1.1	0.7	8	18.50	18.49	92.92	92.97	0.9690	0.9593
17	0.3	0.7	12	18.40	18.41	92.87	92.82	0.9800	0.9897

## Data Availability

Data will be made available on request.

## Supplementary Materials

The following supporting information can be downloaded at: https://www.mdpi.com/article/10.3390/ma16103788/s1. Table S1: Analysis of variance of regression model equation for OAV; Table S2: Analysis of variance of regression model equation for AG; Table S3: Analysis of variance of regression model equation for SV; Equations (S1)–(S3); Figure S1: TG curves of CaCO_3_ before and after modification.
