# Supplementary material for: Surface Modification of CaCO3 by Ultrasound-Assisted Titanate and Silane Coupling Agents
Source: Materials (Basel). 2023 May 17;16(10):3788. doi: 10.3390/ma16103788 (PMC10222999; doi:10.3390/ma16103788)
Supplement: Supplementary file 1 [file materials-16-03788-s001.zip › materials-2382566-supplementary.pdf]

Supporting Information for

Surface Modification of $CaCO_3$ by Ultrasound-Assisted Titanate and Silane Coupling Agents

Peng Cheng [1,2,*], Lei Yang [2], Yuxiong Xie [1] and Yu Liu [1]

[1] School of Civil Engineering, Nanyang Institute of Technology, Nanyang 473004, China
[2] Shaanxi Key Laboratory of Environmental Engineering, Xi'an University of Architecture and Technology, Xi'an 710055, China
* Correspondence: gps_pengcheng@126.com

This file includes：

Equation S1 to S3

Table S1 to S3

Figure S1

Total pages: 4

Taking the OAV, AG, and SV as the response values, the quadratic multinomial regression equations obtained by RSM test are shown as **Equation S1**, **Equation S2**, and **Equation S3**, respectively.

$$OAV = 0.289\text{-}0.153H_1\text{- }0.103H_2\text{- }0.006H_3\text{-}0.001H_1H_2\text{+}0.001H_1H_3\text{-}0.003H_2H_3 + 0.104H_1^2 + 0.088H_2^2 + 0.0004H_3^2 \quad (\mathbf{S1})$$

$$AG = 40.18\text{-}4.22H_1\text{-}33.01H_2\text{+}14.55H_3\text{+}0.14H_1H_2\text{+}1.61H_1H_3\text{+}3.26H_2H_3\text{-}8.94H_1^2 \text{+}0.81H_2^2\text{-}0.91H_3^2 \quad (\mathbf{S2})$$

$$SV = 6.87\text{-}0.86H_1\text{-}2.50H_2\text{-}1.02H_3\text{+}0.09H_1H_2\text{-}0.03H_1H_3\text{+}0.07H_2H_3\text{+}0.77H_1^2\text{+}1.28H_2^2 \text{+}0.05H_3^2 \quad (\boldsymbol{S3})$$

where H_1, H_2 and H_3 are the dosage of HY311 (given in wt% based on $CaCO_3$), the dosage of KH550 (given in wt% based on $CaCO_3$), and the ultrasonication time (min), respectively. The variance analysis of RSM results is shown in **Table S1**, **Table S2**, and **Table S3**, respectively.

Table S1 Analysis of variance of regression model equation for OAV.

Source	Sum of squares	df	Mean square	F-Value	p-Value	
Model	0.0023	9	0.0003	686.84	< 0.0001	Significant
H_1	3.65E-06	1	3.65E-06	9.87	0.0163	
H_2	0.0001	1	0.0001	294.63	< 0.0001	
H_3	6.61E-07	1	6.61E-07	1.79	0.2226	
H_1H_2	1.23E-07	1	1.23E-07	0.3318	0.5826	
H_1H_3	2.72E-06	1	2.72E-06	7.37	0.03	
H_2H_3	0	1	0	57.31	0.0001	
H_1^2	0.0012	1	0.0012	3131.14	< 0.0001	
H_2^2	0.0008	1	0.0008	2265.63	< 0.0001	
H_3^2	9.41E-06	1	9.41E-06	25.49	0.0015	
Residual	2.58E-06	7	3.69E-07			
Lack of Fit	1.39E-06	3	4.64E-07	1.56	0.3309	Not Significant
Pure Error	1.19E-06	4	2.98E-07			
Cor Total	0.0023	16				

Table S2 Analysis of variance of regression model equation for AG.

Source	Sum of squares	df	Mean square	F-Value	p-Value	
Model	102.16	9	11.35	57.38	< 0.0001	Significant
H_1	0.3872	1	0.3872	1.96	0.2045	
H_2	0.8450	1	0.8450	4.27	0.0776	
H_3	0.7080	1	0.7080	3.58	0.1004	
H_1H_2	0.0020	1	0.0020	0.0102	0.9223	
H_1H_3	6.63	1	6.63	33.52	0.0007	
H_2H_3	27.20	1	27.20	137.47	< 0.0001	
H_1^2	8.62	1	8.62	43.57	0.0003	
H_2^2	0.0703	1	0.0703	0.3556	0.5698	
H_3^2	55.20	1	55.20	279.02	< 0.0001	
Residual	1.38	7	0.1978			
Lack of Fit	0.1751	3	0.0584	0.1930	0.8961	Not Significant
Pure Error	1.21	4	0.3024			
Cor Total	103.55	16				

Table S3 Analysis of variance of regression model equation for SV.

Source	Sum of squares	df	Mean square	F-Value	p-Value	
Model	0.4735	9	0.0526	104.46	< 0.0001	Significant
H_1	0.001	1	0.001	2.06	0.1948	
H_2	0.0032	1	0.0032	6.35	0.0398	
H_3	0.0001	1	0.0001	0.2385	0.6402	
H_1H_2	0.0009	1	0.0009	1.79	0.2231	
H_1H_3	0.0027	1	0.0027	5.27	0.0554	
H_2H_3	0.0121	1	0.0121	24.03	0.0017	
H_1^2	0.0646	1	0.0646	128.3	< 0.0001	
H_2^2	0.1772	1	0.1772	351.79	< 0.0001	
H_3^2	0.1665	1	0.1665	330.68	< 0.0001	
Residual	0.0035	7	0.0005			
Lack of Fit	0.0014	3	0.0005	0.8838	0.5213	Not Significant
Pure Error	0.0021	4	0.0005			
Cor Total	0.4770	16				

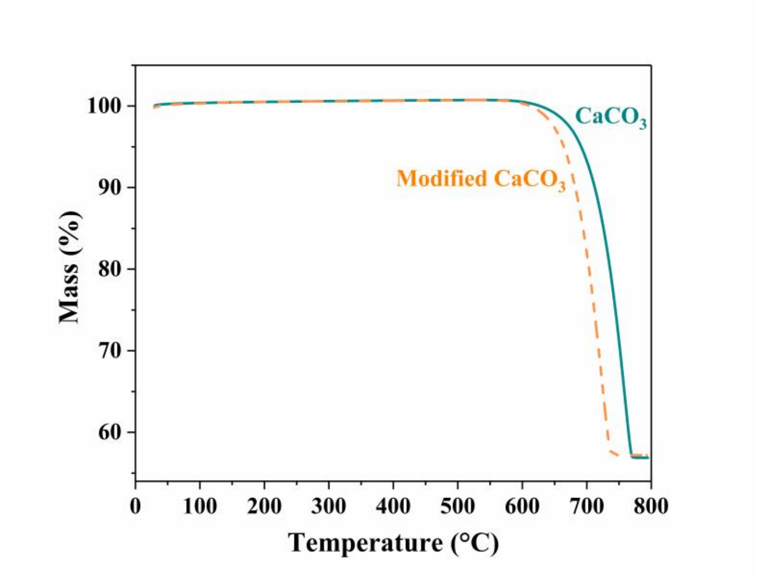

Figure S1. TG curves of $CaCO_3$ before and after modification.
